# Fathers, Childcare and COVID-19

**DOI:** 10.1007/s10691-021-09454-6

**Published:** 2021-05-05

**Authors:** Alice Margaria

**Affiliations:** Department of ‘Law and Anthropology’, Max Planck Institute for Social Anthropology, Halle/Saale, Germany

**Keywords:** COVID-19, Childcare, Family leave, Fathers, Gender equality, Women's employment

## Introduction

Taking a cue from data gathered in spring 2020, this three-part reflection aims to discuss the participation of fathers in childcare during and after the COVID-19 pandemic. Part 1 provides an overview of childcare patterns within heterosexual families during the first wave of lockdowns, as documented by social scientists around the globe. Despite originating in different national contexts, this data seems to indicate a shared human experience: most parents had to juggle paid work with childcare, and fathers spent more time on childcare during spring 2020 than before the pandemic. Part 2 draws inspiration from this data to reconsider family leave policies in view of making ‘active’ fatherhood a widespread and long-term reality. Taking stock of some of the gendered impacts of COVID-19 on women, this reflection concludes by calling for concerted efforts to bring about more equal sharing of childcare responsibilities between men and women. Part 3 considers that if men are to become more responsible for childcare in the long run, it is imperative to prevent the disintegration of decades of hard-won progress in women’s economic opportunities.

## Data and Optimism

Parents have been profoundly impacted by the pandemic. In spring 2020, one of the first and most common measures introduced by governments to slow down the spread of the virus has been the closure of schools and day-care facilities across the world. This situation has generated additional childcare responsibilities for most parents who, on top of that, have often been unable to rely on external sources of support (e.g., grandparents, friends, etc.) due to physical isolation and distancing. Even if some parents began to work from home, the pandemic, and lockdowns in particular, have undeniably intensified pre-existing work-family life conflicts, especially for women with young children (Eurofound 2020, 21–22). Many parents found and still find themselves in the difficult position of taking care of and ‘home-schooling’ their children whilst continuing to perform paid work inside and/or outside the home. What does this mean for the organisation of childcare between mothers and fathers in heterosexual two-parent families?

Abundant evidence (see, e.g., Adams-Prassl et al. 2020; Carlson et al. 2020; Del Boca et al. 2020a; Farré et al. 2020) gathered in spring 2020 documents that the additional childcare responsibilities in the wake of the pandemic have fallen disproportionately on those who used to be the main caretakers prior to the crisis, namely on mothers. In Australia, as a result of COVID-19, mothers spent four extra hours each day on childcare, and fathers only two additional hours (Cooper and Mosseri 2020). In Italy, 61% of working mothers—as opposed to 51% of working fathers—reported undertaking more childcare responsibilities during the first lockdown (spring 2020) than before (Del Boca et al. 2020b). In the UK, data collected between 5 and 11 May 2020 suggests that mothers performed approximately 10 hours of childcare more than fathers per week (Sevilla and Smith 2020, 9).

However, existing data also sheds light on another, more promising aspect of the organisation of childcare during the pandemic: fathers have become more involved than prior to the crisis (see, e.g., Andrew et al. 2020; UN Women 2020; Welsh 2020). In Germany, fathers reported spending 5.3 h/day on childcare in spring 2020, compared to 2.8 h/day in 2019 (Kreyenfeld and Zinn 2021). A comparative survey (Biroli et al. 2020) involving parents in Italy, the UK and the USA in the period of 11–19 April 2020 registered an increase in the number of couples who equally shared childcare activities (17% more in Italy; 8% more in the UK; 11% more in the USA). In Italy, the time used by fathers of children between 3–5 years of age increased from 3 h/day (prior to the lockdown in spring 2020) to 5.5 h/day during the first lockdown (Zannella et al. 2020). Men’s greater (if compared to before the crisis) involvement in childcare was also reported by both men and women in the context of an online survey undertaken in Hungary between 6 and 14 April 2020 (Kováts 2020). Evidence from the Netherlands points in the same direction: as far as childcare is concerned, the gap between mothers' and fathers’ share decreased because fathers did more during the lockdown than before (Yerkes et al. 2020).

This shift on the part of (some) fathers has given rise to (more) optimistic accounts of COVID-19 which emphasise the potential to reduce (instead of only exacerbate) existing gender inequalities. Already in April 2020, on the basis of pre-existing survey data from the USA, Alon et al. (2020a, 2) referred to the expected reallocation of childcare duties between mothers and fathers as a channel through which the pandemic ‘is likely to accelerate changing social norms and expectations’. Additionally, Blaskó et al. (2020, 16), discussing the possible impact of the crisis in an early report published by the European Commission, predicted new opportunities to move towards a more equal distribution of unpaid labour between men and women as a result of the pandemic. This supposition has been maintained in later studies based on data collected in spring 2020. In the Dutch context, Yerkes et al. (2020, 32) consider the ‘new’ childcare patterns following COVID-19 as a ‘potential catalyst for further erosion of traditional role patterns in the division of household and care tasks’. Optimism has gone as far as to make some social scientists wonder whether fathers’ changed behaviour during the pandemic constitutes ‘the first step towards a revolution’ (Zannella et al. 2020) or the sign of a ‘revolution [finally] unstalled’ (Fodor et al. 2020).

One important take-away from the above data on fathers’ behaviour is that being forced or strongly encouraged to stay home and, consequently, being more physically present at home helped men to participate more equally in the care of their children. In other words, changed patterns in paid employment, *in primis*, the unprecedented shift to teleworking caused by the pandemic seems to have contributed to eliminating some of the structural barriers to men’s engagement in childcare and thereby reduced gender imbalances in the distribution of childcare responsibilities between women and men (Carlson et al. 2020). This finds further empirical support in recent studies which documents that the amount of additional childcare provided by men during the crisis is sensitive to, inter alia, their own employment (e.g., Biroli et al. 2020; Mangiavacchi et al. 2020b; Sevilla and Smith 2020), much more than it is for women. Evidence from the UK (Sevilla and Smith 2020, 8), for instance, shows that the allocation of childcare responsibilities has been more equal in households where men were teleworking, had been furloughed or had lost their job.

The increased paternal involvement in childcare is undeniably a welcome development, especially on the heels of a crisis. Yet, can we really expect the recent, more equal sharing of childcare to be sustained over time and to have long-lasting effects in term of gender equality? Some of the (above-mentioned) publications on COVID-19 (see, e.g., Alon et al. 2020a; Andrew et al. 2020) support their optimism by referring to research evidence documenting long-term benefits of fathers’ parental leave taking (see, e.g., Farré and González 2019; Patnaik 2019; Tamm 2019). Given that fathers who use parental leave tend to devote more time to childcare after returning to work as well, the assumption is that also those fathers who have engaged more in childcare during the pandemic will continue to do so in the future.

The perceptions of fathers themselves provide further reason for optimism. An online Canadian survey conducted in May 2020 (Bisby 2020) is a case in point: among 1019 fathers, 40% of the respondents reported positive consequences of COVID-19-related restrictions on their experience as fathers, 52% are more aware of the importance of their role, 60% felt closer to their children, and 48% decided to be more involved as fathers in the future (see also Intentions Consulting 2020). Even so, do we really suppose that, outside of a pandemic context, working fathers (and, more generally, parents) will still feel ‘entitled’ and keen to take advantage of existing opportunities to stay home and take care of their children, and—more generally—to benefit from flexible working arrangements? Moreover, can we presume that employers (and co-workers) have become more aware of and sensitive to the childcare obligations of working parents and, more specifically, of fathers, and their need for flexibility to balance work and family life (in the long run)?

## Reconsidering Family Leave Policies

Whether fathers’ increased participation in childcare will continue over time is largely dependent on the willingness and ability of law and policymakers to capitalise on the changes set in motion by the pandemic. One way to do so is to reconsider existing family leave policies, in particular, those designed for workers who also have childcare duties, with the view of giving fathers a realistic chance to make use of them, and to adopt greater responsibility for childcare more generally. It is a well-established fact that encouraging men’s participation in childcare requires more than contemplating fathers as *potential* beneficiaries of work-life balance by making family leave available to them. The gap existing between fathers’ entitlement to leave policies and their actual uptake is indeed a long-standing and well-documented reality in many contexts (see, e.g., OECD 2016a, 11; Kaufman 2017; Koslowski and Kadar-Satat 2018; Birkett and Forbes 2019). Making men more ‘active’ fathers (Busby and Weldon-Johns 2019, 297) is therefore also a question of assertively encouraging them and, to some extent, even forcing them to make use of existing family leave policies. With this objective in mind, the following paragraphs advance some reflections and proposals expressly concerning COVID-19 family leave, parental leave and paternity leave policies.

In order to support parents and other carers during the pandemic, a very common—and sometimes, the only—policy response has been a flexible approach to working; i.e., to encourage parents to work from home (Koslowski et al. 2020, 10). Some states have taken a more active stance by introducing, inter alia, ‘extraordinary’ family leave policies. As an example, in March 2020, the Italian Government introduced a COVID-19 family leave of 15 days initially, and then increased it to 30 days, for parents of children up to 12 years of age who were unable to work due to childcare responsibilities following the closure of schools and day-care facilities (Decreto Cura Italia 2020, art. 23). This leave was paid at a rate of 50% of previous earnings, could be used by one parent at a time, and most importantly, was accessible only in case of no unemployed or non-working parent, or parent benefiting from income support measures, within the same household.

As argued by Mantione (2020, 126), the Italian COVID-19 family leave has been a missed opportunity for increasing paternal engagement in childcare and, in that vein, to challenge gender inequalities within and outside the home. Gender-disaggregated data are still missing. Nevertheless, the expectation is that the beneficiaries (269,328 parents by 25 May 2020; see INPS 2020) have been mostly women (INAPP 2020, 7). Indeed, the eligibility criterion pertaining to the working status of the other parent means that fathers would not be able to make use of this leave unless the mother participates in paid employment as well. Additionally, even in that scenario, given the lack of a full (or at least higher) payment, the leave is most likely to be taken by mothers, who still generally earn less than men amongst heterosexual couples, in order to mitigate the financial impact on the family income. Initially usable until August 2020, the Italian Government subsequently extended the possibility to make use of this leave until 31 December 2020 in Italy’s most affected regions (where online learning had been re-introduced), stipulating however that only parents who were unable to work from home were eligible (Decreto Ristori bis 2020, art. 13). Suggesting that working from home is to be equated to non-working for the purpose of the COVID-19 family leave reveals an evident lack of forward thinking on the part of the Italian Government. Apart from failing to seize the opportunity to make men more active fathers, this policy does nothing to prevent an imbalanced distribution of the day-to-day childcare duties between mothers and fathers, nor to spare women from a ‘double shift’ during the pandemic. Unsurprisingly, data collected in Italy between 20 April and 9 May 2020 (CGIL 2020, 4) illustrates that, whilst women found working from home ‘more complicated, alienating and stressful’, men (more than women) did not have an obvious preference for working outside the home or from home, or perceived the latter as ‘more stimulating and satisfactory’.

What the Italian experience demonstrates is that if their potential to encourage men’s participation in childcare is to be exploited, COVID-19 family leave policies have to be formulated in a manner which acknowledges and addresses, rather than ignores, women’s and men’s situations as mothers, fathers and workers. To this end, useful guidance can be found in the rich and long-standing debates concerning parental leave policies which have been revived by the COVID-19 situation, mostly as a consequence of the increased paternal engagement in childcare recorded in spring 2020. In recent years, many states have—more or less successfully—reformed their parental leave systems with the aim of increasing men’s use thereof, inter alia, based on the conviction that more equal participation of women in paid employment requires more equal participation of men in the home from the early stages of a child’s life. Let us briefly compare the British and the German policies, whose different design has led to respectively divergent take-up patterns.

In the UK, after the mandatory period of maternity leave, eligible mothers can share the remainder of their entitlement—up to 50 weeks—with their partner.^1^ Thirty-seven weeks of shared parental pay is available at the rate of £151.20/week or 90% of average weekly earnings, whichever is lower (UK Government 2020). Parental leave entitlements are regulated differently in Germany: following a reform introduced in 2007, parents are eligible to 14 months of paid parental leave, 2 months of which are reserved for the mother and 2 for the father. Given that each parent taking leave cannot take less than 2 months, fathers have three options: to take no leave; to take 2 months of paid leave; to take more than 2 months and up to 12 months of paid leave (Tamm 2019, 185). In addition to introducing the so-called ‘daddy months’, the reform has also changed the monthly payment from a flat rate to a benefit equal to 65% of the individual income before childbirth, thus making it considerably higher for most parents (Huebener et al. 2016, 571).

Evidence suggests that shared parental leave (SPL) has gone largely unused in the UK, especially amongst fathers: during the period from April 2015 (when the policy entered into force) to October 2015, between 0.5 and 2% of eligible fathers made use of the new provision (Working Families 2016), and just over 1% of entitled parents took SPL in 2017/2018 (Birkett and Forbes 2018). To the contrary, men’s uptake of parental leave in Germany has been rising since 2007. The share of fathers taking parental leave increased from 3% before the reform to 15% in 2007, and reached 38.8% for births in 2016 (Schober et al. 2020, 288). Further data reveals that, although paid employment patterns remain strongly gendered in Germany, the reformed parental leave system, coupled with other policy reforms—such as the expansion of subsidised childcare for children below 3 years—has also increased mothers’ labour market participation rates (Geyer et al. 2015; Müller and Wrohlich 2020).

What made the UK and German experiences dissimilar is the divergent treatment accorded to fathers’ caring role by their respective parental leave policies. Contrasting with the stated aim to ‘enable working fathers to take a more active role in caring for their children and working parents to share the care of their children’ (Department for Business, Innovation and Skills 2012, 3), the UK SPL regulations de facto prioritise mother’s caring role in various ways (Atkinson 2017, 360; Mitchell 2019, 410–411). Emblematic in this sense is the ‘mediated’ nature of fathers’ entitlement to SPL: the mother, once her eligibility is ascertained, allows the transfer of the leave to the father (Mitchell 2019, 410). On the other hand, the German policy assertively promotes paternal engagement in childcare not only by granting fathers an autonomous right to parental leave, but especially by reserving some portion of (well-)paid parental leave to fathers.

In more concrete terms, this comparison shows that low levels of pay combined with the lack of non-transferable periods of leave are likely to leave the status quo unaltered, or even further entrench it, whereas increasing individual non-transferrable entitlements to paid leave and payment rates is likely to encourage fathers to make use of parental leave policies. The latter contributes to reducing some of the obstacles often faced by men as fathers (Karu and Tremblay 2018), to cite a few: financial barriers due to a potential loss of income; gender norms and cultural expectations which see mothers as the primary caretakers; and perceived low support from the workplace (OECD 2016b; Eurofound 2019; Kolowski et al. 2020, 33). Recent developments at the level of the European Union (EU) also support and, to some extent, even demand parental leave policies which actively promote fathers’ participation in childcare. In particular, the 2019 Work-life Balance Directive, to be adopted by Member States by 2022, requires at least two non-transferrable months of parental leave.^2^ Notwithstanding its limitations (Caracciolo di Torella and Masselot 2010, chapter 5), the hope is that this Directive will assist Member States in reorienting their family leave policies more firmly towards ‘active fatherhood’ (Busby and Weldon-Johns 2019, 297).

This leads us to some final reflections on paternity leave policies as an additional means of bringing fathers into the realm of childcare. Another ‘big novelty’ (Collomber and Math 2019) brought by the 2019 Work-Life Balance Directive is the introduction of at least 10 days of paternity leave around childbirth (art. 4), compensated at the level of sick pay at least (art. 8(2)). Granting EU paternity-specific rights to fathers conveys powerful messages: that fathers are able to take care of newborns—as much as mothers—and that fathers need a job-protected period of paid leave around childbirth, even if for different reasons than mothers. Yet, at the national level, more can be and has already been done through mandatory paternity leave policies.

As a case in point, in Portugal, fathers can take up to 25 working days of paternity leave (called ‘fathers-only parental leave’), 20 of which are mandatory, paid at 100% of previous earnings with no upper limit (Kolowski et al. 2020, 472). If, according to some, forcing fathers to take some leave may constitute an excessively strong infringement of personal autonomy, it seems to have contributed to some important change at the societal level in Portugal both in terms of public opinion, where fathers’ caring role has gained improved recognition, and men’s use of it (Ferreira 2020). Not only has the percentage of fathers taking mandatory paternity leave increased, but most fathers who take the mandatory leave also make use of the optional (paternity leave) days (Wall et al. 2020, 482). Further evidence also shows a rise in fathers’ uptake of ‘initial parental leave’ (formerly ‘maternity leave’), which consists of either 120 days at 100% of earnings or 150 days at 80% of earnings, and can be shared between the parents, with the exception of the first 6 weeks after childbirth which are reserved and mandatory for the mother (Wall et al. 2020, 481).

Of course, fathers’ increased uptake has not been the result of mandatory paternity leave alone. Rather, it is the outcome of two decades of policy efforts directed to improve work-life balance, children’s well-being, and gender equality—in particular through enhancing fathers’ leave entitlements (Wall and Leitão 2017, 45). Apart from extending the length of paid paternity leave, the latest reform (2009) introduced a ‘sharing bonus’ scheme in accordance with which an extra 30 days of fully compensated leave is made available should the father take 4 weeks or more of initial parental leave on his own after the first 6 weeks reserved for the mother. This has proven to be a very successful measure: the number of fathers sharing the leave increased from 0.6% in 2009 (before the reform) to 20% in 2010, 1 year after the reform, and continues to increase albeit at a slower pace (Wall et al. 2020, 481).

That being said, the transformative potential of mandatory paternity leave is undeniable. Forcing fathers to take (some) paternity leave facilitates choice by establishing a minimum period of leave which is not open to negotiation and discussion (Fredman 2014, 451; Rosenblum 2020), and depending on the context, it may initiate or boost an already-ongoing process of rethinking fathers’ caring role. Fathers, other carers, employers, and society at large become aware that childcare is a core responsibility of being a father. If accompanied by incentives for fathers to stay home after the mandatory paternity leave period, it can give rise to longer leave periods for fathers, more ‘fathers on leave alone’ (O’Brien and Wall 2017), and presumably, fathers’ greater responsibility for childcare.

Of course, merely replicating measures or policies which have been successful in a specific country is no guarantee of similar behavioural change elsewhere (Karu and Tremblay 2018; Birkett and Forbes 2019, 218). Nonetheless, on a broader level, all the above-mentioned policy experiences seem to suggest that more assertive family leave policies are needed to make men more active fathers. Not the mere availability of leave to fathers, but their actual uptake of leave under circumstances which actually enable them to participate more equally in childcare will promote more egalitarian practices at home and at work (Suk 2019, 463). Each in their own context-specific way, policy and law makers are therefore confronted with the arduous, yet essential, task of striking a balance between acknowledging that women’s and men’s caring experiences have been and continue to be different overall, and challenging and disestablishing the traditional gendered division of labour.

## Conclusion: The Reality, the Other Side of the Coin

Besides posing new challenges, the pandemic offers the opportunity to reconsider pre-existing laws and policies to enable working fathers to take on a more equal responsibility for childcare, and to challenge gender inequalities in general. It is therefore important not to lose the momentum, to build on the (more) promising data outlined above in order to revise existing family policies and make them more assertive in increasing fathers’ uptake and contribution to childcare.

At the same time, however, it is imperative not to lose sight of the other side of the coin: women’s participation in paid employment. The possibility of overcoming a gendered division of labour is indeed—at any time: before, during, or after the pandemic—also dependent upon women’s position in the job market and their contribution to the family income. The same data collected in spring 2020 confirms that the partner’s employment situation exercised great influence over the share of additional childcare assumed by fathers, more than by mothers (Andrew et al. 2020; Del Boca et al. 2020a; Farré et al. 2020; Mangiavacchi et al. 2020a). In the UK, for instance, in families where the mother stopped working in paid employment during the lockdown while the father continued, the mother did twice as much childcare as her male partner (Andrew et al. 2020, 4). If the situation was reversed (i.e., the father stopped working while the mother continued), childcare was shared equally between parents, while the mother continued to do 5 hours of paid work per day (Andrew et al. 2020, 4).

Attending to women’s position in paid employment following the start of the pandemic, the situation appears far less rosy and opportunity-rich. The majority of sectors most affected by the pandemic predominantly employ women (International Labour Organisation 2020, 9). This confirms in growing unemployment rates affecting women more severely than men (ILO 2021). In addition to those who lost their jobs, many women (and their families) have made the ‘pragmatic’ choice to leave the labour market and stop looking for a job in order to secure care for their children and, more generally, navigate current circumstances and related uncertainties (e.g., see ISTAT 2020). Analyses of past financial crises have indicated that their negative gendered effects on, inter alia, women’s participation in paid employment may become particularly apparent after 3 years, and persist even 7 years after the end of a crisis (Blanton et al. 2019, 943). Therefore, even those women who are still in paid employment are bound to suffer long-term disadvantages as a consequence of the current pandemic recession. The prediction is indeed that the additional childcare responsibilities women have undertaken following COVID-19 are likely to inhibit work and career progression for working mothers, in a context where gender pay and promotion gaps remain a significant obstacle to gender equality (Alon et al. 2020b).

If seen in this wider context, the time spent on childcare by some fathers during the pandemic is a drop in the ocean. To enable such drops to swell into rivers of change, investing in facilitating access to and actual use of work-family policies by men must be far more convincing. Women’s economic opportunities should be a priority after the pandemic to prevent them falling further behind. The success of any reform of family leave policies will be contingent on a strong commitment to bringing about a more equal sharing of paid work and childcare responsibilities between men and women (in a different context: Busby and Weldon-Johns 2019, 298). For family leave policies to trigger even a small transformation in the home, they need to be part of an effective, comprehensive gender equality strategy to redress the wider social and economic consequences of the pandemic. This means investing more in, inter alia, the care infrastructure in view of its employment-generating potential for women and the female-dominated sectors of employment—especially those most adversely affected by the pandemic (Klatzer and Rinaldi 2020).

## Footnotes

1 Shared Parental Leave Regula

2 Direcve (EU) 2019/1158 of the European Parliament and of the Council of 20 June 2019 on worklife balance for parents and carers and repealing Council Direcve 2010/18/EU, art. 5.
